# Comparative clinical efficacy of single-incision thoracoscopic bullectomy combined with C-shaped electrocautery pleurodesis vs. chemical pleurodesis in the management of spontaneous pneumothorax

**DOI:** 10.3389/fsurg.2024.1480240

**Published:** 2024-11-12

**Authors:** Bin Zhong, Qiyong Wu, Ming Zhang

**Affiliations:** Department of Thoracic Surgery, The Affiliated Changzhou No. 2 People’s Hospital of Nanjing Medical University, Changzhou, China

**Keywords:** spontaneous pneumothorax, single-incision, thoracoscopic surgery, pleurodesis, pain score

## Abstract

**Objective:**

This study aims to observe the clinical efficacy of single-incision thoracoscopic bullectomy combined with C-shaped electrocautery pleurodesis compared to traditional iodine chemical pleurodesis in the treatment of patients with spontaneous pneumothorax.

**Methods:**

A total of 128 patients with spontaneous pneumothorax who underwent surgical treatment at our institution from January 2021 to December 2022 were selected. Patients were categorized into the study group (*n* = 65) and the control group (*n* = 63) based on the surgical method used. The study group received C-shaped pleura cautery for pleurodesis, while the control group underwent traditional iodine chemical pleurodesis. Surgical and clinical outcomes were evaluated, and recurrence rates within one year postoperatively were compared. Serum C-reactive protein (CRP) levels and visual analog scale (VAS) scores were measured at different time points.

**Results:**

No significant differences were observed between the two groups regarding surgical duration, intraoperative blood loss, or postoperative hospital stay (*p* > 0.05). However, the study group demonstrated significantly lower serum CRP levels and VAS scores on postoperative days 1 and 2, as well as reduced drainage volumes compared to the control group (*p* < 0.05). The recurrence rates of pneumothorax within one year postoperatively did not differ significantly between the groups (*p* > 0.05).

**Conclusion:**

Single-incision thoracoscopic bullectomy combined with C-shaped electrocautery pleurodesis is an effective treatment for spontaneous pneumothorax. Compared to traditional iodine chemical pleurodesis, this method causes less pleural irritation, results in lower postoperative pain and drainage, and facilitates patient recovery, making it a safe and reliable option for the management of spontaneous pneumothorax.

## Introduction

Spontaneous pneumothorax (SP) is a common thoracic condition characterized by the accumulation of air in the pleural space, leading to lung collapse. Epidemiological studies indicate that the incidence of SP is estimated at 17–24 cases per 100,000 in males and 1–6 cases per 100,000 in females (1). Treatment options for SP include observation, needle aspiration, tube thoracostomy, and surgical intervention, with the latter being reserved for more severe or recurrent cases.

Surgical options typically involve bullectomy, which is the resection of large pulmonary blebs, as they are often the source of air leaks. However, the recurrence rate of pneumothorax following simple bullectomy remains high, ranging from 17% to 54% (1, 2). This necessitates the need for additional procedures, such as pleurodesis, which can help obliterate the pleural space and reduce the risk of recurrence (2, 3). Various pleurodesis techniques have been established, including chemical pleurodesis using agents like iodine and doxycycline (4), as well as mechanical pleurodesis methods (5, 6). Each approach has its advantages and challenges, offering different outcomes in terms of efficacy, safety, and side effects (7–9).

Recently, we developed a novel pleurodesis technique aimed at minimizing postoperative complications and enhancing patient recovery. This new method involves the use of electrocautery to perform multi-point cauterization of the intercostal pleura, creating multiple C-shaped rings (10). This technique promotes effective pleural adhesion while potentially reducing intraoperative bleeding and postoperative pain. The purpose of this study is to evaluate the clinical efficacy of this technique in conjunction with single-incision thoracoscopic bullectomy compared to the traditional iodine chemical pleurodesis in the management of SP. By examining surgical outcomes, recurrence rates, and postoperative recovery, we aim to establish a more effective protocol for treating this challenging condition.

## Data and methods

### General information

This study included 128 patients with SP who underwent surgical treatment at our institution between January 2021 and December 2022. Patients were subjected to single-incision thoracoscopic bullectomy, with subsequent division into two groups based on the type of pleurodesis performed: the study group (*n* = 65) received C-shaped electrocautery pleurodesis, while the control group (*n* = 63) underwent traditional iodine chemical pleurodesis.

#### Inclusion criteria

1. Patients diagnosed with SP via computerized tomography (CT) of the chest, without significant hemothorax.
2. Normal cardiac, hepatic, and renal function tests, presenting clear surgical indications.
3. No history of chest trauma, tuberculosis, or pneumonia.
4. Postoperative pathological examination consistent with pulmonary blebs.

#### Exclusion criteria

1. Secondary pneumothorax.
2. History of prior thoracic surgery.
3. Coexisting dysfunction of vital organs prohibiting surgery.
4. Coagulation disorders.

### Ethical considerations

This study was approved by the Medical Research Ethics Committee of Changzhou No. 2 People's Hospital [Ethics Approval No: (2020) YLJSA301].

### Surgical technique

All procedures were performed using single-port thoracoscopic bullectomy. Patients were placed under general anesthesia with double-lumen endotracheal intubation, allowing for single-lung ventilation. A 3 cm incision was made along the anterior axillary line at the 4th intercostal space, serving as both the thoracoscopic and working port. The chest cavity was filled with saline to facilitate examination, and the anesthesiologist assisted in lung inflation. Using the thoracoscope, the site of the air leak and the location of the blebs were identified. Once confirmed, single-lung ventilation was resumed, and a one-time use cutting and closure device (Johnson & Johnson Medical Devices, Shanghai, China, model SC 45 A) was employed to excise the target bleb and any surrounding weakened lung tissue. Reinforcement sutures were placed in the remaining lung tissue as needed. Upon confirming the absence of air leaks at the resection sites, pleurodesis was performed, followed by the placement of a drainage tube (Pacific Medical Supplies Co., China, 24 F) through the working port. The chest was closed in layers, and the skin was sutured. Postoperative management included standard antimicrobial therapy, analgesia, fluid replacement, and symptomatic support.

In the study group, C-shaped cautery pleurodesis was performed by applying an electrocautery pen to ablate the parietal pleura from the 2nd to the 6th intercostal spaces. Multiple circular areas of coagulation formed distinct C-shaped rings, ensuring superficial application limited to the pleura without damaging intercostal nerves or major blood vessels. For the control group, 2% iodine tincture (National Drug Standard No: H 44023918, Guangdong Hengjian Pharmaceutical Co., 2% × 500 ml) was applied via sterile gauze to the visceral pleura until hyperemia was noted, indicating sufficient pleural irritation. After 1minute, residual fluids were aspirated, and drainage tubes were placed similarly.

#### Observation indicators

1. Comparison of surgical indicators, including operative time, intraoperative blood loss, postoperative drainage volume, and length of hospital stay.
2. Collection of peripheral venous blood samples preoperatively and on postoperative days 1 and 2 to assess serum C-reactive protein (CRP) levels.
3. Evaluation of pain severity using the Visual Analog Scale (VAS) preoperatively and at 24 h (day 1) and 48 h (day 2) postoperatively. All patients received standard analgesic therapy consisting of Loxoprofen Sodium Tablets (60 mg, orally, twice daily). VAS scoring was characterized as follows: 0 indicates no pain, 10 indicates the worst pain, <3 signifies good analgesic effect, and ≥5 indicates poor analgesic effect.
4. Monitoring recurrence of pneumothorax within one year postoperatively.

### Statistical analysis

Data were processed using SPSS version 26.0. Continuous variables with normal distribution were expressed as mean ± standard deviation $\left(\bar{x}\pm s\right)$, while those not following a normal distribution were denoted as median (interquartile range). Comparisons of mean values between groups were carried out using independent samples *t*-tests for normally distributed data and nonparametric tests for non-normally distributed data. Repeated measures analysis was used for comparisons across multiple time points, while categorical data were expressed as percentages and analyzed using the *χ*^2^ test. A significance level of *α* = 0.05 was set, with *p* < 0.05 indicating statistical significance.

## Results

### Patient characteristics

The baseline characteristics of the patients in both groups were comparable, with no significant differences observed in terms of gender and age (*p* > 0.05), as summarized in Table 1.

**Table 1:** Comparison of general data of patients between the two groups.

Group	Gender		Age (years)
	Male	Female	
C-shaped cautery pleurodesis (*n* = 65)	59 (90.77%)	6 (9.23%)	26.0 (13)
Chemical pleurodesis (*n* = 63)	56 (88.89%)	7 (11.11%)	27.0 (10)
t/*χ*^2^ value	0.124		
*P* value	0.725	0.864	

### Surgical outcomes

The surgical parameters, including operation time, intraoperative blood loss, postoperative drainage volume, length of hospital stay, and one-year recurrence rates, were compared between the two groups, as shown in Table 2. Notably, the postoperative drainage volume in the C-shaped cautery pleurodesis group was significantly less than that of the chemical pleurodesis group, with statistical significance (*p* < 0.05). However, no significant differences were found between the groups concerning operation time, intraoperative blood loss, and length of hospital stay (*p* > 0.05). Both groups experienced recurrences of pneumothorax, with the recurrence rate in the C-shaped cautery pleurodesis group being lower than that in the chemical pleurodesis group; yet, this difference was not statistically significant (*p* > 0.05).

**Table 2:** Comparison of surgical indexes between the two groups.

Group	Operation time(min)	Bleeding volume (ml)	Drainage volume (ml)	Postoperative hospital stay(d)	Recurrence
C-shaped cautery pleurodesis (*n* = 65)	46.25 ± 7.54	16.86 ± 5.39	156.31 ± 42.03	3 (1)	1 (1.54%)
Chemical pleurodesis (*n* = 63)	45.97 ± 9.64	33.11 ± 9.14	263.65 ± 73.63	3 (0)	4 (6.35%)
t/*χ*^2^ value	0.181	−12.201	−10.088		0.899
*P* value	0.856	<0.001	<0.001	0.144	0.343

**Figure 1:**
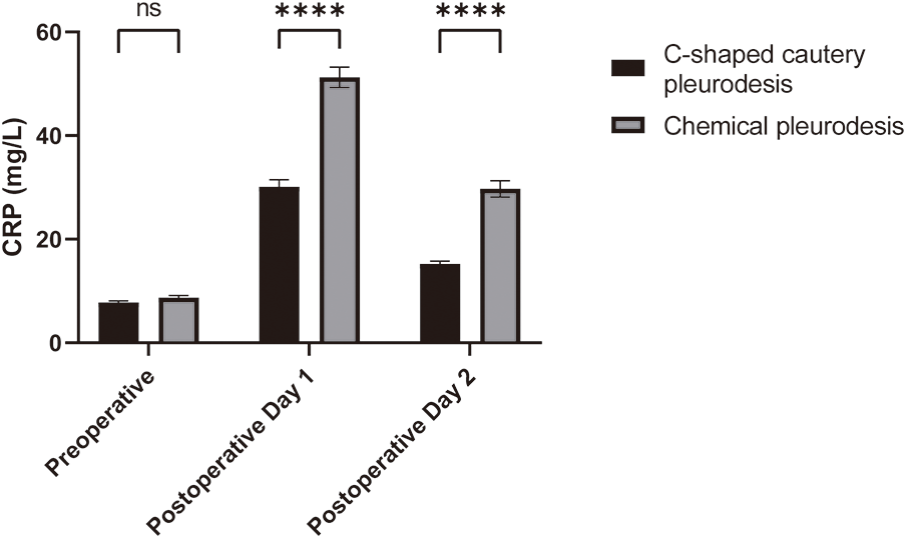
Comparison of CRP levels in the patients of the two groups. Compared with the chemical pleurodesis group, ***p < 0.001.

Comparison of CRP and VAS Levels: The comparison of serum C-reactive protein (CRP) levels and Visual Analog Scale (VAS) scores between the two groups before surgery and on postoperative days 1 and 2 is presented in Table 3. Prior to the surgery, there were no significant differences in serum CRP levels between the two groups (*p* > 0.05). On postoperative days 1 and 2, however, the serum CRP levels in the C-shaped cautery pleurodesis group were significantly lower than those in the chemical pleurodesis group (*p* < 0.05), as illustrated in Figure 1. Repeated measures analysis indicated a significant difference in CRP levels within each group over time (*p* < 0.05).

**Table 3:** Comparison of CRP levels and VAS scores between the two groups.

Group	CRP levels (mg/L)			VAS scores		
	Preoperative day	Postoperative day 1	Postoperative day 2	Preoperative day	Postoperative day 1	Postoperative day 2
C-shaped cautery pleurodesis (*n* = 65)	7.76 ± 2.72	30.12 ± 10.91	15.22 ± 4.59	0 (1)	4 (1)	2 (1)
Chemical pleurodesis (*n* = 63)	8.74 ± 3.26	51.25 ± 15.56	29.70 ± 12.62	0 (0)	4 (1)	2 (1)
t/*χ*^2^ value	−1.844	−8.871	−8.573			
*P* value	0.068	<0.001	<0.001	0.901	<0.001	<0.001
*P* value (Repeated measures analysis)	<0.001			<0.001		

**Figure 2:**
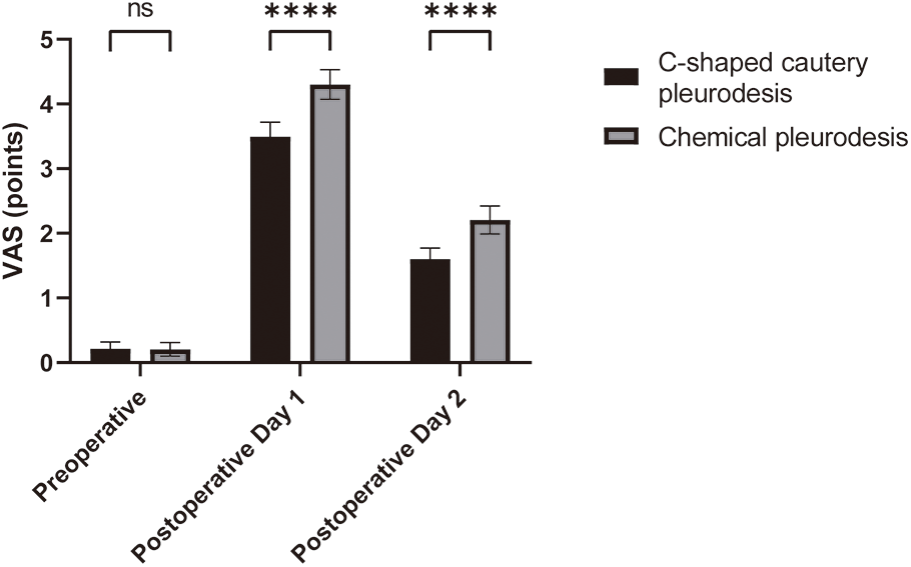
Comparison of VAS scores in the patients of the two groups. Compared with the chemical pleurodesis group, ***p < 0.001.

Additionally, no significant differences in VAS scores were observed between the two groups before surgery (*p* > 0.05). On postoperative days 1 and 2, the VAS scores of patients in the C-shaped cautery pleurodesis group were significantly lower than those of the chemical pleurodesis group (*p* < 0.05), as depicted in Figure 2. Repeated measures analysis confirmed a significant difference in VAS scores between the groups over time (*p* < 0.05).

In summary, while surgical outcomes such as operative time and hospital stay showed no significant differences, the C-shaped cautery pleurodesis group benefited from reduced postoperative drainage and showed notably lower inflammation and pain levels indicated by CRP and VAS scores. The recurrence rates, although numerically lower in the C-shaped cautery pleurodesis group, did not reach statistical significance.

## Discussion

This study provides important insights into the outcomes of single-incision thoracoscopic bullectomy for spontaneous pneumothorax, specifically comparing the efficacy of C-shaped electrocautery pleurodesis with traditional iodine chemical pleurodesis. Our findings suggest significant improvements in several postoperative metrics when using the C-shaped cautery technique, indicating a shift towards more effective and streamlined surgical management for this condition.

A principal observation was the reduced postoperative drainage volume in the C-shaped cautery group, which is an important parameter as excessive drainage can prolong hospital stays and introduce potential complications. Lower drainage volumes are likely a result of more effective pleurodesis achieved through cautery, which might facilitate a quicker and more robust adhesion formation, thereby reducing effusion. Previous literature has suggested that the choice of pleurodesis technique can directly influence the rates of pleural fluid accumulation (11, 12). Therefore, our results underscore the importance of surgical technique selection, as minimizing drainage translates to both enhanced patient comfort and reduced healthcare resource utilization.

Additionally, the significantly lower serum CRP levels observed in the cautery group on postoperative days 1 and 2 signal a less pronounced inflammatory response associated with this technique. Elevated CRP levels are a recognized biomarker for inflammation and can correlate with postoperative complications, including infections and prolonged recovery periods (13–15). The reduced inflammatory response in the C-shaped cautery group may also reflect a more minimally invasive approach that leads to less tissue trauma, thus promoting a faster healing process. These findings align with existing studies that emphasize the advantages of low-trauma techniques in surgical applications, suggesting that techniques promoting physiological healing can significantly enhance recovery.

Pain management is another critical component of postoperative recovery that can significantly affect patient satisfaction and overall outcomes (12). Our analysis demonstrated that patients who underwent C-shaped pleura cautery pleurodesis reported lower VAS pain scores on postoperative days 1 and 2. Effective pain control is essential, as unrelieved pain can hinder mobility, increase the risk of complications, and prolong recovery time (16, 17). The lower pain levels observed with C-shaped pleura electrocautery pleurodesis may be explained by several factors. The pleura's dense innervation and the introduction of air during pneumothorax often lead to significant pain. In contrast, chemical pleurodesis, which uses strong agents like iodine, tends to cause more intense pain, exacerbated by a greater inflammatory response in the pleural cavity. These differences warrant further investigation to clarify the underlying mechanisms.

While recurrence rates of spontaneous pneumothorax did not reach statistical significance, there was just a numerical trend favoring the C-shaped cautery group. Recurrence of pneumothorax remains a complex issue, often dependent on individual patient factors, including underlying lung pathology and genetic predisposition (18, 19). The existing literature firmly establishes that chemical pleurodesis is an effective method for preventing the recurrence of pleural effusion. The lack of significant differences in recurrence rates observed in our study may suggest the non-inferiority of C-shaped pleura cautery pleurodesis. However, we also recognize that the small sample size and short follow-up period may influence these outcomes. To address this limitation, we emphasize the necessity for future studies with larger sample sizes and longer follow-up periods to enhance the generalizability of our findings.

One notable limitation of our study is its retrospective design, which may introduce biases inherent in data collection and analysis. A small sample size also restricts the generalizability of the findings. To strengthen our conclusions, larger-scale, multicenter prospective studies are warranted. These studies should further investigate not only immediate postoperative outcomes but also longer-term follow-up data regarding recurrence rates and quality of life metrics.

In conclusion, C-shaped pleura cautery pleurodesis appears to provide favorable outcomes in single-incision thoracoscopic bullectomy for spontaneous pneumothorax, marked by reduced drainage volumes, lower inflammatory markers, and improved pain management. These findings advocate for a reevaluation of pleurodesis techniques in the surgical treatment of spontaneous pneumothorax, reinforcing the importance of methodological precision in surgical practice. Outreach for further research is essential to fully elucidate the long-term implications associated with this promising technique.

## Data Availability

The raw data supporting the conclusions of this article will be made available by the authors, without undue reservation.
